# The impact of pancreatic cancer screening on life expectancy: A systematic review of modeling studies

**DOI:** 10.1002/ijc.34379

**Published:** 2022-12-14

**Authors:** Brechtje D. M. Koopmann, Amir‐Houshang Omidvari, Iris Lansdorp‐Vogelaar, Djuna L. Cahen, Marco J. Bruno, Inge M. C. M. de Kok

**Affiliations:** ^1^ Department of Public Health Erasmus MC University Medical Center Rotterdam The Netherlands; ^2^ Department of Gastroenterology & Hepatology Erasmus MC University Medical Center Rotterdam The Netherlands

**Keywords:** high‐risk, mathematical model, pancreatic cancer, screening

## Abstract

Evidence supporting the effectiveness of pancreatic cancer (PC) screening is scant. Most clinical studies concern small populations with short follow‐up durations. Mathematical models are useful to estimate long‐term effects of PC screening using short‐term indicators. This systematic review aims to evaluate the impact of PC screening on life expectancy (LE) in model‐based studies. Therefore, we searched four databases (Embase, Medline, Web‐of‐science, Cochrane) until 30 May 2022 to identify model‐based studies evaluating the impact of PC screening on LE in different risk populations. Two authors independently screened identified papers, extracted data and assessed the methodological quality of studies. A descriptive analysis was performed and the impact of screening strategies on LE of different risk groups was reported. Our search resulted in 419 studies, of which eight met the eligibility criteria (mathematical model, PC screening, LE). Reported relative risks (RR) for PC varied from 1 to 70. In higher risk individuals (RR > 5), annual screening (by imaging with 56% sensitivity for HGD/early stage PC) predicted to increase LE of screened individuals by 20 to 260 days. In the general population, one‐time PC screening was estimated to decrease LE (2‐110 days), depending on the test characteristics and treatment mortality risk. In conclusion, although the models use different and sometimes outdated or unrealistic assumptions, it seems that PC screening in high‐risk populations improves LE, and that this gain increases with a higher PC risk. Updated model studies, with data from large clinical trials are necessary to predict the long‐term effect of PC screening more accurately.

## Graphical abstract

What's new?

Robust evidence on the effectiveness of pancreatic cancer screening is lacking, as published studies are mostly cohort studies, limited in size and duration. Here, the authors performed a systematic review to evaluate the impact of pancreatic cancer screening on life expectancy as determined in model‐based studies. The results show that, while it could be detrimental in the general population, pancreatic cancer screening in high‐risk populations may increase life expectancy. Updated model studies, with data from large clinical trials, are necessary to predict the long‐term effect of pancreatic cancer screening more accurately.

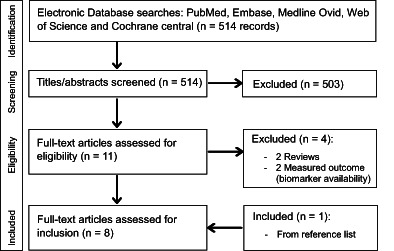

**Abbreviations**
AGA: American Gastroenterological Association
CI: confidence interval
CT: computed tomography
EUS: endoscopic ultrasound
FPC: familial pancreatic cancer
ICER: incremental cost effectiveness ratio
LE: life expectancy
LYG: life years gained
MRI: magnetic resonance imaging
NA: not applicable
NNS: number needed to screen
NNT: number needed to treat
PanIN: pancreatic intraepithelial neoplasia
PC: pancreatic cancer
PRISMA: Preferred Reporting Items for Systematic reviews and Meta‐Analyses
PROSPERO: Prospective Register of Systematic Reviews
RR: relative risk
SB‐IPMN: Side Branch—Intraductal Papillary Mucinous Neoplasm
Sens.: sensitivity
Spec.: specificity

## 1 INTRODUCTION

Pancreatic cancer (PC) is mostly diagnosed at an incurable stage.^1^ Early detection by screening might improve survival, but robust evidence on the effectiveness of PC screening is lacking, as published studies are cohort studies, limited in size and duration. Ideally, large clinical trials with decades of follow‐up would provide solid evidence on the efficacy of screening. Since such studies are unlikely to be concluded promptly, mathematical models can be used to predict long‐term outcomes, using short‐term indicators from observational studies.

Mathematical models, also known as decision‐analytic models, can simulate health outcomes of individual patients or a population under a variety of scenarios. They can be used to estimate the consequences of distinct screening scenarios under varying circumstances. Several types of mathematical models exist, such as decision trees, Markov models and microsimulation models.

These models all have a similar principle of decision processes. These processes relate to potential events that can occur. For example, an individual can be at a certain risk to develop a precursor lesion. Based on this risk, a precursor lesion will or will not develop during his or her lifetime. If a precursor lesion develops, the associated cancer risk will determine the chance that it will progress to a malignancy. Decision processes can be relatively simple, such as a decision tree, or more complex, such as a microsimulation model. In a decision tree the risk of events and states of nature is diagramed over a fixed time horizon. Markov models simulate a hypothetical cohort of individuals through health states over time. A microsimulation model simulates individual life histories and tracks the past health states and future events for each individual.^2^ These decision‐analytic models can inform policy makers on optimal screening strategies for several cancer types. Also, they can estimate the cost‐effectiveness and harms and benefits of a screening program.^3^, ^4^ In the past, these models have been used to quantify the effects of screening for cancer such as breast, colon, cervix, and lung cancer in a population.^3^, ^4^, ^5^, ^6^ Mathematical modeling is, for example, used to translate colorectal cancer screening trial results (that showed screening effectiveness in a controlled setting) to real world health outcomes for the total population and a longer time period.^7^, ^8^

In the last decade, several mathematical models on PC screening have been developed to evaluate the efficacy of screening with a variety of screening modalities, screening strategies, and risk populations.^9^, ^10^, ^11^, ^12^, ^13^ The objective of the current systematic review is to gain insight into the estimated effects of these mathematical models pertaining life expectancy (LE).

## 2 METHODS

This systematic review was executed according to the Preferred Reporting Items for Systematic reviews and Meta‐Analyses (PRISMA) guideline. The review protocol is available in PROSPERO (CRD42020168804). We searched four electronic databases (Embase, Ovid Medline, Web of Science, Cochrane central) for studies published before 30 May 2022. The search strategies used for each database are presented in the Supplementary Materials (Data S1).

### 2.1 Inclusion and exclusion criteria

We searched for studies that used a mathematical model to evaluate the effect of PC screening on LE in different PC risk groups. Mathematical models were defined as models using given input to simulate a decision process with the help of algorithms (eg, simulation, Markov, microsimulation or decision tree models). Our search was limited to papers written in English and involving human subjects. We excluded studies that evaluated the effect of PC treatment. We also excluded reviews, in vitro studies, case reports and letters.

### 2.2 Study selection and data extraction

After removing duplicates, two reviewers (B.K. and A.O) independently screened titles, abstracts and full texts for potentially eligible studies. In case of disagreement, discrepancies between the two reviewers were discussed, and if no consensus was reached, a third reviewer (IdK) joined to settle. The reference lists of included studies were scanned to identify potential additional studies. The following information was extracted (if available): authors, publication year, model type, model input (source), simulated population (PC risk level, gender), screening method (diagnostic method, test characteristics, screening interval, start‐, stop age, complication risk), treatment (type, effect, complication risk, morbidity), screening effect (LE), Life years gained (LYG), number needed to screen (NNS), number needed to treat (NNT). If detailed results of a certain analysis were not available, we contacted the corresponding author to get access to the results.

### 2.3 Quality assessment

We adapted the ISPOR‐AMCP‐NPC Questionnaire^14^ to assess the relevance and credibility of each modeling study according to the following criteria: (I) Validation; (II) Bias due to the study design; (III) Limitations in data sources; (IV) Appropriateness of the model analysis; (V) Reporting bias; (VI) Interpretation bias; and (VII) Conflict of interest. The risk of bias for each domain was rated as low, high, or unclear. The checklist is available in the Supplementary Materials (Data S1). This assessment provided us with information on the quality of the included studies, which is important when results are translated to clinical practice.

### 2.4 Data synthesis and analysis

We performed a descriptive analysis and summarized the effect of PC screening on LE. We compared the screening effect on LE for the different risk groups in the included studies (general population, high‐risk group, different risk levels/mutation carriers).

## 3 RESULTS

### 3.1 Models

**FIGURE 1:**
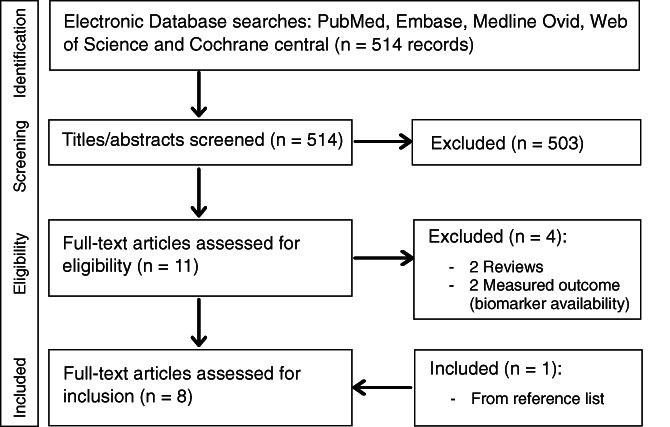
PRISMA flow chart

Our search resulted in 514 articles, of which 11 underwent full text review after title and abstract screening (Figure 1). Four papers were excluded based on study type (review) and/or model outcome (LE was not reported). One paper was found by evaluation of reference lists. Altogether, eight articles met the inclusion criteria, published between 2003 and 2021.

Table 1 provides an overview of the included studies. Six were performed in the United States, one in Italy and one in the Netherlands. The simulated populations varied from general populations, to high‐risk groups (such as BRCA2 gene mutation carriers or kindreds of familial pancreatic cancer patients [FPC] or individuals with pancreatic cystic lesions), to PC patients. In this latter group the effect of detection in an earlier stage on LE was evaluated. The models encompass five Markov models, one Monte Carlo model, one Microsimulation model and one decision tree.^9^, ^10^, ^11^, ^12^, ^13^, ^15^, ^16^, ^17^ Three studies from a single research group used the same base model, which was adjusted for different research questions.

**TABLE 1:** Overview included studies

Study	Modeling aim	Modeling method	Simulated population	Outcome
Rulyak, 2003^12^	Cost‐effectiveness of EUS screening in FPC kindreds	Decision tree	Members of familial pancreatic cancer kindreds	Endoscopic screening was cost‐effective, with an incremental cost‐effectiveness ratio of 16 885/life‐year saved. Screening was more cost‐effective as the probability of dysplasia increased and as the sensitivity of the test increased. Expected gain in LE was 38 years for a cohort of 100 people
Weinberg, 2010^15^	Assist patients and physicians with understanding how their decisions concerning pancreatic cysts affect overall survival/QoL	Markov model	Pancreatic cyst (SB‐IPMN located in head)	Initial pancreaticoduodenectomy was the dominant strategy to maximize overall survival for any cyst >2 cm, regardless of age or comorbidities. “Do nothing” maximized quality of life for all cysts <3 cm in patients aged <75, when measuring quality adjusted survival
Pandharipande, 2015^9^	Identify when, from the standpoint of RR, one time screening is effective in high‐risk individuals for PC	Markov model	RR for PC from 1 to 70	One time MR imaging screening in average risk group of 100 000 men at age 50 identifies 2375 low risk cysts, 159 high‐risk cysts and 56 cancers. Resulting in 39 cancer deaths averted and a net LE loss of 3 days). If the PC risk exceeds 2.4 (men) or 2.7 (women) there was a gain in LE
Pandharipande, 2015^10^	Compare effectiveness of different PC screening strategies in BRCA2 mutation carriers, from standpoint of life expectancy	Markov model	BRCA2 mutation carriers (with or without FDR with PC)	One time screening at age 50 resulted in a LE gain of 3.9 days for the BRCA2 cohort. The gain was higher with more FDRs with PC. Annual screening resulted in a LE loss of 12.9 days for BRCA2 mutation carriers. BRCA2 carriers with 2 FDRs gained 20.6 days with annual screening
Cucchetti, 2016^16^	Verify survival benefit obtained from a hypothetical screening where a 20,30 or 50% reduction of PC stage was obtained	Monte Carlo Simulation	Pancreatic cancer patients	Mean expected LE for PC patients was 13 months. When a hypothetical screening reduced stage III/IV with 30‐50% this LE was: 14‐15.9 months
Peters, 2018^11^	Gain insight into the natural history of PanIN and to assess the potential of screening	Markov model	General population	Lifetime probability for PanIN1 to progress to PDAC (1.5%). Duration of this progression: 33.6 years. A hypothetical perfect test for PanIN 3 detection and treatment could provide a maximum, average LE gain of 40 days
Raphel, 2018^17^	Determine the effect of patient age and comorbidity on LE benefits associated with SB‐IPMN follow‐up	Markov model	Pancreatic cyst (SB‐IPMN)	The LE benefit of SB‐IPMN follow‐up is 5.3‐6.4 months for healthy 60 year old individuals. The effect on LE was limited in case of 80 year old individuals and coexistence of severe comorbidity
Koopmann, 2021^13^	Analyze the impact of relevant uncertainties on the effect of PC screening in high‐risk individuals	Markov model	High‐risk individuals	Screening reduced PC mortality in all modeled scenarios. The reduction depended strongly on natural disease course (progressive vs indolent and faster progressive lesions). The impact of test sensitivity was much smaller. The NNS was impacted most by PC risk

The study aims of all included articles differed. Some focused on efficacy of screening in different risk groups, while others focused on the natural disease course of PC and its precursor lesions.

Besides variations in screen frequency and starting age, models used different surveillance tests; varying between magnetic resonance imaging (MRI), Endoscopic Ultrasound (EUS), Computed tomography (CT), a combination of imaging tests or a hypothetical test. Two papers assumed a 100% sensitive test for PanIN3 and PC detection.^11^, ^17^ Although unrealistic, this model provides insight in the maximal gain of screening in a hypothetical optimal scenario.

A really low sensitivity of 56% for detection of HGD/early stage PC with MRI was described by Pandharipande et al^9^, ^10^ used a sensitivity of 56% for detection of HGD/early stage PC with MRI. This sensitivity was based on pooled data from six clinical studies dating from 2009 to 2012. Cases (resected and/or clinical PC) within these studies were classified into true‐positive (resectable PC), false‐negative (advanced PC), true‐negative and false‐positive (surgery + no PC). Because of a paucity of available data on cystic lesions, similar test characteristics were assumed for high risk cysts.

Rulyak et al used an EUS sensitivity of 90% for the detection of dysplasia based on expert opinion.^12^ An abnormal EUS was defined as two or more of the following abnormalities: heterogenous parenchyma with echogenic foci, hypoechoic nodules, hyperechoic main duct wall or discrete masses. A positive test led to a diagnostic Endoscopic Retrograde Cholangiopancreatography (ERCP) for confirmation. Koopmann et al used preliminary data from a large surveillance cohort to assess a stage dependent sensitivity (60%‐99%) for a combined EUS/MRI test.^13^ An overview of the extracted data from the included models is presented in Table 2.

**TABLE 2:** Overview extracted data from included studies

	1	2	3	4	5	6	7	8
First author + publication year	Rulyak et al,^12^ 2003	Weinberg et al,^15^ 2010	Pandharipande et al,^9^ 2015	Pandharipande et al,^10^ 2015	Cucchetti et al,^16^ 2016	Peters et al,^11^ 2018	Raphel et al,^17^ 2018	Koopmann et al,^13^ 2021
Journal	Gastrointestinal endoscopy	Gastroenterology	Radiology	Ebiomedicine	Pancreas	Pancreatology	Radiology	International journal of cancer
Country	USA	USA	USA	USA	Italy	USA	USA	The Netherlands
Model type	Decision Tree	Markov based clinical Nomogram	Markov model	Markov model	Monte Carlo Simulation	Markov model	Markov model	Markov model
Population	Members of FPC kindreds	Pancreatic cystic lesions, male and female (cyst: 0.5 to >3 cm)	General population to high risk group (both male and female)	BRCA2 mutation carriers (with/without FDRs)	1000 PC patients	General population	Pancreatic cyst (SB‐IPMN), male and female, different comorbidities	High risk group (7.5% life time risk)
PC Risk	18% lifetime risk^a^	1%	RR 0‐70 for PC	RR 3.5 or greater	100%^b^	RR 0	Low risk incidental findings	7.5% lifetime risk
Screen method	EUS (on indication + ERCP)	CT or EUS (± FNA)	MRI scan	MRI (combined with EUS)	Hypothetical test	Hypothetical test	Hypothetical imaging test	EUS and MRI
Screen sens. (sens. analysis)	90% for detection of pancreatic dysplasia	80%‐86%	56% (0.25‐1.0) For detection of cyst and early stage cancer. No PanIN.	56% (0.5‐1.0) For detection of cyst and early stage cancer. No PanIN.	NA	100% for detection of PanIN3	100% for malignant lesions	Disease stage dependent: 60‐99% (±10%)
Screen spec. (sens. analysis)	90%	99%	97% (0.5‐1.0)	97% (0.9‐1.0)	NA	100%	NA	99% (±5 and 10%)
Screen test complication	Pancreatitis: 5.1% (0.3‐8.2)	Death from EUS‐FNA: 0.01%	NA	NA	NA	NA	NA	NA
Screening interval	One time		One time	Annual and one time	One time	One time and continuous	Annual	Annual and 5 yearly
Start/stop age (sens. analysis)	50	65, 75, 85	50 (40, 60, 70)	50‐80	NA	50, 60 or 70	60,80	
Treatment	Surgery (total pancreatectomy)	Surgery	Surgery	Surgery	Surgery	Surgery	Surgery	Surgery
Treatment complication risk (sens. analysis)	Mortality risk: 3% (1%‐5%)	Mortality risk: 2%‐6.4% Chronic compl: 19.5%	Mortality risk: 2% (0%‐10%)	Mortality risk: 1% (0%‐2.5%)	NA	Mortality risk: 0 and 2%	Mortality risk: 0% (2%‐4%)	Mortality risk: 3% (5%)

a Prevalence of dysplasia (0.20) x progression probability to PC given the presence of dysplasia (0.90) = 0.18.

b Analysis performed on individuals already diagnoses with PC. Consequences of earlier detection were evaluated.

### 3.2 Screening in a high‐risk population

Four studies reported on the effect of screening in different high‐risk populations.^9^, ^10^, ^12^, ^13^ The effect of PC screening on LE depended on the relative risk (RR) for PC of the screened population and the test characteristics (Table 3). Pandharipande et al reported that one‐time MRI screening (sensitivity 56%, specificity 97% for pancreatic cyst and early stage cancer) in 50‐year‐old individuals with a 2.4 to 4.5 RR for PC (eg, BRCA2) led to a negligible gain in LE of 3.9 to 5.8 days per simulated individual. Annual MRI screening even reduced LE by 1.3 to 12.9 days in this risk group. When higher risk individuals (RR 6.4% or 7.5% life time risk) were screened annually, a gain in LE of up to 158 days was predicted, depending on test performance.^13^

**TABLE 3:** Effect of different PC screening strategies on life expectancy

Risk group	Screening method	Screening interval	Age	Treatment mortality (%)	Effect on LE (days) per simulated individual	Reference
General population	MRI (sens. 56%, spec. 97%, for detection of cyst and early stage PC. No PanIN)	One time	40	2	−5.1 to −5.8^a^	9
			50	2	−3.3 to −4.0^a^	
				10	−27 to −31^a^	
				1	2 to 3^a^	
			60	2	−2.2 to −2.9^a^	
			70	2	−1.5 to −1.8^a^	
	MRI (sens. 25%, spec. 97%, for detection of cyst and early stage PC. No PanIN)	One time	50	2	−4 to −5^a^	
	MRI (sens. 56%, spec. 50%, for detection of cyst and early stage PC. No PanIN)	One time	50	2	−94 to −110^a^	
	MRI (sens. 56%, spec. 100%, for detection of cyst early stage PC. No PanIN)	One time	50	2	2	
	MRI (sens. 100%, spec. 97%, for detection of cyst and early stage PC. No PanIN.	One time	50	2	−2	
	Hypothetical perfect test for PanIN3	Continuous	50	0	40	11
				2	37	
		One time	50	0	7.1	
				2	6.7	
			60	0	10	
				2	9.3	
			70	0	7.9	
				2	7	

Inherited increased risk for PC						
RR 2.4‐4.5	MRI (sens. 56%, spec. 97%, for detection of cyst and early stage PC. No PanIN)	One time	50	1	3.9 to 5.8	10
		Annual	50	1	−12.9 to −1.3	
RR 6.4/life time risk 7.5%	MRI (sens. 56%, spec. 97%, for detection of cyst and early stage PC. No PanIN)	One time	50	1	9.1	10
		Annual (50–80)	50	1	20.6	
	MRI + EUS (sens. per disease stage [60–99%], spec. 90%)	Annual	50	3	120.4‐158.4^b^	13
	MRI + EUS (sens. per disease stage [60‐99%], spec. 90%)	5‐yearly	50	3	48.6‐90.4^b^	
RR 12 (PC life time risk 18%)	EUS (sens. 90%, for detection of pancreatic dysplasia)	One time	50	3	138.7	12
RR 30‐32	MRI (sens. 56%, spec. 97%, for detection of cyst and early stage PC. No PanIN)	One time	50	1	31.5	10
				2	65 to 71^a^	9
		Annual	50	1	260	10
RR 70	MRI (sens. 56%, spec. 97%, for detection of early stage PC)	One time	50	2	160 to 181^a^	9
PC	Hypothetical screening reducing stage III/IV cancer with 20%	NA	NA	NA	54.7	16
	Hypothetical screening reducing stage III/IV cancer with 30%	NA	NA	NA	82.1	
	Hypothetical screening reducing stage III/IV cancer with 50%	NA	NA	NA	152	

Pancreatic cyst				Test mortality (%)		
Cyst (1‐3 cm^c^)	CT/MRI (Detecting HGD + PC. Probability true pos. = 0.80, true neg. = 0.99)	Annual	65	0	17 to 439	15
			75	0	9 to 50	
			85	0	3 to 54	
	EUS ± FNA (Detecting HGD + PC. Probability true pos. = 0.86, true neg. = 0.99)	Annual	65	0.01	18 to 445	
			75	0.01	10 to 55	
			85	0.01	4 to 59	
SB‐IPMN	Imaging	Annual	60	0	161 to 195	17
			80	0	69	

*Note*: The effect on LE is visible in days lost or gained per simulated individual entering screening. Morality risk is either caused by screentest (EUS) or by treatment (surgery after positive screentest).

Abbreviations: CT, computed tomography; EUS, endoscopic ultrasound; HGD, high grade dysplasia; MRI, magnetic resonance imaging; Neg., negative; PanIN, Pancreatic Intraepithelial Neoplasia; PC, pancreatic cancer; Pos., positive; RR, relative risk; SB‐IPMN, Side Branch—Intraductal Papillary Mucinous Neoplasm; Sens., sensitivity; Spec., specificity.

a Effect on life expectancy for men and women.

b In this model two pathways are simulated, one with only progressive lesions and one with indolent and faster progressive lesions. A larger effect on LE was seen in the progressive‐only pathway.

c LYG are presented in years per patient in ranges correlated with cyst size (1 cm less gain, 3 cm, more gain in LE).

Rulyak et al reported a gain of 139 days per simulated individual when EUS (sensitivity 90% for pancreatic dysplasia) was performed once at age 50 in family members of familial pancreatic cancer kindreds. They assumed individuals in this risk group had a 20% risk of having dysplasia and a 90% risk of progression to PC when having dysplasia. This resulted in an estimated 18% risk of PC, given a 1.5% PC life‐time risk in the general population, which can be compared to a RR of 12.

A higher PC risk (ie, RR of 30) also led to a higher LE gain (31.5 for one time and 260 days for annual screening). The effect of one‐time MRI screening was highest in individuals with a RR of 70. Their LE increased by 160 days for men and 188 days for women.^9^

Cucchetti et al evaluated the effect on LE in case of an earlier PC diagnosis after hypothetical screening.^16^ They evaluated the impact of stage reduction on LE for different stages. The mean LE without screening was 13 months (395.2 days) from diagnosis. The 3‐ and 5‐year survival rates were 14.1% and 3.8%, respectively. When a hypothetical screening program reduced stage III/IV PC by 20%, 30% or 50%, the mean LE increased to 450, 477 and 547 days, respectively.^16^ After adjustment for lead‐time, LE was 426, 444 and 483 days.

### 3.3 Screening individuals with pancreatic cystic lesions

Two studies evaluated the effect of screening in individuals with pancreatic cysts.^15^, ^17^ Weinberg et al evaluated the effect of annual screening with computed tomography (CT)/MRI or endoscopic ultrasound (EUS) in patients with different size cystic lesions suggestive of Side Branch—Intraductal Papillary Mucinous Neoplasm (SB‐IPMN), specifically in the pancreatic head. Their results show that the effect on LE was larger when screening started at age 65, as compared to starting at 75 or 85, both for CT/MRI and EUS screening. The effect on LE increased with cyst size. In case of annual screening with CT starting at age 65, a gain of 17 days was achieved for cystic lesions of 1 cm. If the cystic lesion was 3 cm, the LE gain for the same screening scenario was 439 days per screened individual. When individuals aged 85 received annual screening with CT, the average LE gain was 3 days in case of a 1 cm cystic lesion and 54 days in case of a 3‐cm cyst. Similar results were seen for screening with EUS (Table 3).

Raphel et al evaluated the effect of surveillance in patients with SB‐IPMN and analyzed the effect on LE for individuals with different comorbidity levels.^17^ For healthy men and women, the LE benefit was 161 and 195 days, respectively. In 80‐year‐old individuals with severe comorbidity, surveillance had less effect on LE (36 days).

### 3.4 Screening in the general population

Two studies evaluated the effect of PC screening in the general population.^9^, ^11^ The first reported that one‐time screening either at age 40, 50, 60 or 70 with MRI (sensitivity 56%, specificity 97% for HGD pancreatic cyst and early stage cancer) resulted in a LE loss of 1.5 to 5.8 days per screened individual. A lower test specificity of 50% resulted in an even higher loss of LE (94‐100 days). An increase in test sensitivity to 100% hardly effected LE (LE loss of 2 vs 4 days with 56%‐sensitivity).

The second study evaluated the maximum effect of surveillance in the general population with a hypothetical perfect test for pancreatic intraepithelial neoplasia (PanIN).^11^ When a 2% treatment mortality was considered, one time screening improved LE by a mere 6.7 to 9.3 days per screened individual. In the hypothetical optimal scenario where screening with this perfect test was continuous (eg, daily), a 40‐day gain in LE was found for each simulated individual.

### 3.5 Risk of bias

**FIGURE 2:**
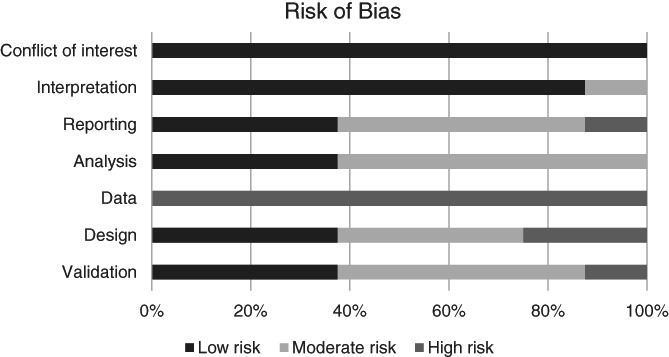
Risk of bias

Especially in the field of PC, where data are scarce and evidence is based on small studies, the risk of bias regarding input or target data is relatively high. Figure 2 provides an overview of the risk of bias of the included articles per item. Bias assessment per study is available in the Supplementary Materials (Data S1).

## 4 DISCUSSION

This systematic review evaluates model‐based analyses on the effect of PC screening on LE. PC screening in the general population led to decreased LE in most analyses, as screening benefits were outweighed by false positive tests and surgical mortality risks. For higher‐risk individuals, screening was more beneficial, but this conclusion strongly depends on model assumptions such as test characteristics. The effect of surveillance on LE seemed small in the slightly increased risk group (RR < 4.5). However, in this analysis, an unrealistic low test sensitivity (56%) for HGD/early stage PC were used in this analysis. As a result, these predictions likely underestimate the potential effect of screening. In individuals at higher risk (RR 6.4‐60, eg, CDKN2A), both annual and one‐time screening improved LE, even with a poor screening test. Although most papers were based on outdated and unrealistic assumptions, they do show that PC screening can be worthwhile. In the future, predictions from these models will improve when they are updated with reliable input data from ongoing long‐term studies.

In higher‐risk individuals (RR: 30), the expected median LE gain of 260 days for annual PC screening seems worthwhile. In comparison, colorectal cancer screening with an annual FIT test in the general population is estimated to increase LE by 89 days per screened individual.^18^ For lung cancer, 30 days are gained by annual screening with a low dose CT of 50 to 75‐year old males who smoke.^19^

However, LE gain is not the only parameter to determine effectiveness of a screening program. Other factors, such as impact on quality of life, but also harms and costs should be considered. Moreover, morbidity associated with the screening methodology or the surgical treatment (pancreatic fistula, exocrine pancreatic insufficiency and diabetes) was not taken into account in most studies, although this is highly relevant.

The effect of screening on LE varies within the models because of different model assumptions, for instance on the natural history. In two models, PC could only evolve from “pancreatic dysplasia” or adenomas.^12^, ^15^ Distinct precursor types such as IPMN and PanIN, that can progress from LGD to HGD into cancer, were not defined. The models that did simulate both precursors in separate pathways assumed that 90% of PC developed from PanIN and 10% from cystic lesions.^9^, ^10^, ^11^ If the proportion of cyst derived cancers is actually higher, as some studies indicate,^20^, ^21^, ^22^ these models would also underestimate the efficacy of screening.

Input on cyst prevalence is needed to simulate a population at (increased) risk for pancreatic precursor lesions and PC.^9^, ^10^, ^11^, ^13^ The reported cyst prevalence in the general population varies from 2.6% to 55%. This range is caused by differences in imaging techniques, populations and cyst definition (size and type). Pandharipande et al used an imaging based cyst prevalence of 4.6% at age 50 as a calibration target. An increased cyst prevalence was assumed in the models that simulated a higher risk group, as is consistent with literature.^23^, ^24^

Next to differences in natural history, the models incorporated different screen scenarios and assumed different screen tests characteristics. Test sensitivity for cystic lesions and PC varied from 56% to 99%. Also, most models used the same sensitivity for all disease stages, which is unrealistic. Pandharipande et al assumed a sensitivity of 56% for both cystic lesions and early stage cancer. When we evaluate more recent publications on PC screening in high risk individuals, a sensitivity of 70% to 75% for HGD/early stage cancer seems more realistic.^23^, ^25^, ^26^ A lower test sensitivity leads to an underestimation of the screening effect. However, with frequent screening, the effect of test sensitivity on life years saved is relatively small.^13^

Also, the use of a composite sensitivity for the detection of both HGD and early stage PDAC is a limitation. Overbeek et al published results of PC screening in high risk individuals, and showed that none of the 14 resected lesions harbored HGD. This shows how difficult detection of HGD is and that using the sensitivity for PDAC likely overestimates test performance for HGD.

Ideally, each detected lesion on imaging is pathologically confirmed. However, a lesion without worrisome features on imaging is not resected, and thus no pathological confirmation is available. In surveillance studies, only resected lesions can be compared head‐to‐head, causing selection bias.

One model evaluated the effect of a hypothetical test that detects all PanIN3 lesions. Under this ideal (but for now unrealistic) assumption, one‐time PC screening led to a LE gain, even in the general population, although that gain was still low compared to other cancer screening programs. However, in clinical practice, we fail to detect PanIN lesions with current imaging modalities, let alone determine their dysplastic grade. However, this unrealistic scenario provides useful information on the upper limit of effectiveness of screening, and the maximal benefit in life expectancy it can provide.

It is very difficult to distinguish low from high grade dysplasia, based on imaging. Although cyst characteristics can give some guidance, a discrepancy between the imaging based diagnosis, and the true pathological state is observed.^27^ In the model of Pandharipande et al,^9^ the proportion of high‐risk cystic lesions was estimated using a proxy as a calibration target: the proportion of patients with cysts who underwent subsequent pancreatic surgery was considered “high‐risk” (5/84). However, as we know from surgical series, not nearly all individuals with high‐risk features harbor actual high‐grade dysplasia or PC. A large meta‐analysis showed a pooled sensitivity of 0.67 (95% Confidence Interval [CI] 0.64‐0.70) and a specificity of 0.64 (95% CI 0.62‐0.66) for the Fukuoka guideline to detect advanced neoplasia (HGD or PC). Similar results were seen for the American Gastroenterological Association (AGA) guideline (pooled sensitivity of 0.59 [95% CI 0.52‐0.65] and specificity of 0.77 [95% CI 0.74‐0.80]).^27^

The simulated screening strategies vary from one time to annual, to 5‐yearly screening. Multiple studies evaluate the effect of one time screening at age 50. The relevance of this strategy is questionable given the PC peak incidence at age 60 to 70 years. However, the relevance also depends on duration of disease stages and test characteristics. One time screening at age 60 or 65 would be an interesting alternative scenario to study.

Assumed treatment mortality varied from 0% to 10%. Recent literature shows a mortality risk of 1% to 3% for a Whipple procedure and 1% to 2% for pancreatic tail resections.^28^, ^29^ Thus, the study that assumed a 10% treatment mortality may have overestimated the harms of screening, resulting in an even larger loss in LE.

Finally, of the models that evaluated PC screening in high‐risk individuals, only one accounted for the increased risk to die from other prevalent malignancies in mutation carriers.^10^ The assumption that high‐risk individuals have an average probability to die of other causes in the other models seems inappropriate and leads to an overestimation of life‐years gained. Importantly, mathematical models use observational data to simulate disease progression and evaluate effects of screening. Compared to other cancer models, observational PC data on the natural history and test characteristics are scant. This is a source for bias and may hamper the reliability of decision‐analytic models.

Despite these limitations, the results of this systematic review on PC screening are important for clinical practice and future research. They show the potential and limitations of screening on LE in different PC risk groups and emphasize the importance of selecting the right population for screening and the need for better screening tests to correctly identify and classify pancreatic precursor lesions. Especially for PanIN lesions, there is a lot to gain. For a screening program to be successful, given the current screening tests, the focus should be on individuals from the highest risk groups. Consensus guidelines advice screening for individuals with a 5% life time risk for PC.^30^ This PC risk greatly depends on test characteristics. Future model updates, based on more reliable input data from large and long‐term studies should focus on establishing this.

Screening is always associated with lead and length‐time bias. In this review, only one study reported on this.^16^ They estimated a lead‐time bias of 23 to 64 days, which has considerable impact, given the short survival. They corrected for this bias by using the tumor volume doubling time, after which the NNS encompassed the harmful threshold. Other studies evaluating the effect of PC screening did not account for this bias. Thus, the true effect of surveillance in these groups is even smaller.

The effect of PC screening in ongoing clinical trials is mainly based on early detection of cancer rather than resection of HGD cyst, as published data shows that the number of resected HGD lesions is low (n = 0,^31^ n = 0,^23^ n = 10^23^, ^25^). Precursor lesions that have been resected were mainly LGD. As the majority of LGD cysts will never progress to PC (given the high cyst prevalence and low PC incidence), the impact of a resected LGD cyst on life expectancy will be low and possibly negative because of treatment complications. Trials also show that patients with early detected and resected PC still have a limited life expectancy.^23^ The 5 year survival rate of T1 patients was reported to be 30.6%.^32^ For future research, focus should therefore lay on the detection and treatment of HGD precursor lesions. The detection of early stage cancer, even with annual screening, seems challenging and screen detected early stage PC remains associated with a decreased LE.

Unfortunately, current screen tests are underperforming in the detection of high‐grade dysplastic precursor lesions. Despite the existence of multiple risk prediction tools, it remains difficult to classify cysts and determine their grade of dysplasia correctly, based on imaging.^27^ Also, test specificity for PanIN lesions is nowhere near 97%, while this lesion is believed to account for 50% to 90% of all PC. Thus, it is important to search for new and better screen tests. In this regard biomarkers hold promise, not only to detect early cancer, but also to search for timely identification of (high grade dysplastic) precursor lesions.^33^

In conclusion, even though most included papers used outdated and unrealistic assumptions, our study shows that screening can be effective in high‐risk individuals. The extend of this effect depends on PC risk, test characteristics and screen frequency. Long lasting clinical trials are needed to gain more insight in the natural history of PC and the true test sensitivity by disease stage. Such input data will improve the predictive performance of PC screening models.

## AUTHOR CONTRIBUTIONS

**Brechtje D. M. Koopmann**: Conceptualization, data curation, formal analysis, methodology, writing original draft. **Amir‐Houshang Omidvari**: Data curation, formal analysis, methodology, writing original draft. **Iris Lansdorp‐Vogelaar**: Supervision, review & editing. **Djuna L. Cahen**: Funding, supervision, review & editing. **Marco J. Bruno**: Funding, supervision, review & editing. **Inge M. C. M. de Kok**: Funding, Methodology, supervision, review & editing. The work reported in the paper has been performed by the authors, unless clearly specified in the text.

## FUNDING INFORMATION

Brechtje D. M. Koopmann, Djuna L. Cahen, Marco J. Bruno and Inge M. C. M. de Kok received funding from the MLDS (nongovernmental organization, national gastrointestinal disease association, nonprofit). Brechtje D. M. Koopmann, Amir‐Houshang Omidvari, Iris Lansdorp‐Vogelaar, Djuna L. Cahen, Marco J. Bruno and Inge M. C. M. de Kok have nothing to disclose.

## CONFLICT OF INTEREST

The authors declare no potential conflict of interests.

## ETHICS STATEMENT

No ethical approval was needed for this systematic review because data from previous published modeling studies with simulated individuals was retrieved and analyzed.

## Supporting information


**Data S1**. Supporting Information.

## Data Availability

All data used in this systematic review is available in the original papers that were included.
