# Supplementary material for: The impact of pancreatic cancer screening on life expectancy: A systematic review of modeling studies
Source: Int J Cancer. 2022 Dec 14;152(8):1570–80. doi: 10.1002/ijc.34379 (PMC10107819; doi:10.1002/ijc.34379)
Supplement: Supplementary file 1 — Data S1. Supporting Information. [file IJC-152-1570-s001.pdf]

Supplementary material

THE IMPACT OF PANCREATIC CANCER SCREENING ON LIFE EXPECTANCY: A SYSTEMATIC REVIEW OF MODELLING STUDIES

Brechtje D.M. Koopmann, MD, Amir H. Omidvari, MD, PhD; Iris Lansdorp-Vogelaar, PhD; Djuna L. Cahen, MD, PhD; Marco J. Bruno, MD, PhD; Inge M.C.M. de Kok, PhD

Table of contents

1) Literature Search
2) ISPOR-AMCP-NPC Questionnaire
3) Bias assessment

1 - Literature Search

Embase

('pancreas tumor'/exp OR (((pancrea*) NEAR/6 (carcinoma* OR adenocarcinoma* OR adenoma* OR cystadenoma* OR neoplas* OR tumo* OR cancer* OR lesion*)) OR ipmn OR (Intraduct* NEAR/6 Papillary NEAR/6 Mucin* NEAR/6 Neoplas*)):ab,ti,kw) AND ('screening'/de OR 'cancer screening'/de OR 'mass screening'/de OR 'surveillance'/de OR 'disease surveillance'/de OR 'health survey'/de OR 'early diagnosis'/de OR 'early cancer diagnosis'/de OR (screening OR surveillan* OR 'health survey' OR (early NEAR/3 (diagnos* OR detect*))):ab,ti,kw) AND ('model'/de OR 'theoretical model'/de OR 'structural model'/de OR simulation/de OR 'Computer Simulation'/de OR 'Patient Simulation'/de OR 'disease simulation'/de OR 'algorithm'/de OR 'mathematical model'/de OR (algorithm* OR ((mathematical* OR statistical*) NEAR/3 model*) OR simulation* OR microsimulation*):ab,ti,kw) NOT ([animals]/lim NOT [humans]/lim) NOT ([Conference Abstract]/lim) AND [English]/lim

Ovid Medline

(Pancreatic Neoplasms/ OR Carcinoma, Pancreatic Ductal/ OR Pancreatic Intraductal Neoplasms/ OR (((pancrea*) ADJ6 (carcinoma* OR adenocarcinoma* OR adenoma* OR cystadenoma* OR neoplas* OR tumo* OR cancer* OR lesion*)) OR ipmn OR (Intraduct* ADJ6 Papillary ADJ6 Mucin* ADJ6 Neoplas*)).ab,ti,kw.) AND (Mass Screening/ OR Diagnostic Screening Programs/ OR Public Health Surveillance/ OR Population Surveillance/ OR Health Surveys/ OR Early Detection of Cancer/ OR Early Diagnosis/ OR (screening OR surveillan* OR health survey OR (early ADJ3 (diagnos* OR detect*))).ab,ti,kw.) AND (Models, Theoretical/ OR Models, Structural/ OR Computer Simulation/ OR Patient Simulation/ OR Algorithms/ OR Models, Statistical/ OR (algorithm* OR ((mathematical* OR statistical*) ADJ3 model*) OR simulation* OR microsimulation*).ab,ti,kw.) NOT (exp animals/ NOT humans/) AND english.la.

Web of Science

TS=(((((pancrea*) NEAR/5 (carcinoma* OR adenocarcinoma* OR adenoma* OR cystadenoma* OR neoplas* OR tumo* OR cancer* OR lesion*)) OR ipmn OR (Intraduct* NEAR/5 Papillary NEAR/5 Mucin* NEAR/5 Neoplas*))) AND ((screening OR surveillan* OR "health survey" OR (early NEAR/2 (diagnos* OR detect*)))) AND ((algorithm* OR ((mathematical* OR statistical*) NEAR/2 model*) OR simulation*OR microsimulation*))) AND DT=(article)

Cochrane CENTRAL

((((pancrea*) NEAR/6 (carcinoma* OR adenocarcinoma* OR adenoma* OR cystadenoma* OR neoplas* OR tumo* OR cancer* OR lesion*)) OR ipmn OR (Intraduct* NEAR/6 Papillary NEAR/6 Mucin* NEAR/6 Neoplas*)):ab,ti,kw) AND ((screening OR surveillan* OR 'health survey' OR (early NEAR/3 (diagnos* OR detect*))):ab,ti,kw) AND ((algorithm* OR ((mathematical* OR statistical*) NEAR/3 model*) OR simulation* OR microsimulation*):ab,ti,kw)

2 - ISPOR-AMCP-NPC Questionnaire (1)

Relevance

1 Is the population relevant?

- Are the demographics similar?
- Are risk factors similar?
- Are behaviours similar?
- Is the medical condition similar?
- Are comorbidities similar?

2 Are any critical interventions missing?

- Does the intervention analyzed in the model match the intervention you are interested in?
- Have all relevant comparators been considered?
- Does the background care in the model match yours?

3 Are any relevant outcomes missing?

- Are the health outcomes relevant to you considered?
- Are the economic end points relevant to you considered?

4 Is the context (settings and circumstances) applicable?

- Is the geographic location similar?
- Is the health care system similar?
- Is the time horizon applicable to your decision?
- Is the analytic perspective appropriate to your decision problem?

Credibility

Validation

1 Is external validation of the model sufficient to make its results credible for your decision?

- Has the model been shown to accurately reproduce what was observed in the data used to create the model?
- Has the model been shown to accurately estimate what actually happened in one or more separate studies?
- Has the model been shown to accurately forecast what eventually happens in reality?

2 Is internal verification of the model sufficient to make its results credible for your decision?

- Have the process of internal verification and its results been documented in detail?
- Has the testing been performed systematically?
- Does the testing indicate that all the equations are consistent with their data sources?
- Does the testing indicate that the coding has been correctly implemented?

3 Does the model have sufficient face validity to make its results credible for your decision?

- Does the model contain all the aspects considered relevant to the decision?
- Are all the relevant aspects represented and linked according to the best understanding of their characteristics?
- Have the best available data sources been used to inform the various aspects?
- Is the time horizon sufficiently long to account for all relevant aspects of the decision problem?
- Are the results plausible?
- If others have rated the face validity, did they have a stake in the results?

Design

4 Is the design of the model adequate for your decision problem?

- Was there a clear, written statement of the decision problem, modelling objective, and scope of the model?
- Was there a formal process for developing the model design (e.g. influence diagram, concept map)?
- Is the model concept and structure consistent with, and adequate to address, the decision problem/objective and the policy context?
- Have any assumptions implied by the design of the model been described, and are they reasonable for your decision problem?
- Is the choice of model type appropriate?
- Were key uncertainties in model structure identified and their implications discussed?

Data

5 Are the data used in populating the model suitable for your decision problem?

- All things considered, do you agree with the values used for the inputs?
- Did the approaches to obtaining and processing the data inputs meet the criteria from their corresponding questionnaires?

Analysis

6 Were the analyses performed using the model adequate to inform your decision problem?

7 Was there an adequate assessment of the effects of uncertainty?

Reporting

8 Was the reporting of the model adequate to inform your decision problem?

- Did the report of the analyses provide the results needed for your decision problem?
- Was adequate nontechnical documentation freely accessible to any interested reader?
- Was technical documentation, in sufficient detail to allow (potentially) for replication, made available openly or under agreements that protect intellectual property?

Interpretation

9 Was the interpretation of results fair and balanced?

<u>Conflict of Interest</u>

10 Were there any potential conflicts of interest?

11 If there were potential conflicts of interest, were steps taken to address these?

Reference:

1) Jaime Caro J ED, Kan H, Kaltz C, Patel B, Eldessouki R, Briggs AH; . ISPOR-AMCP-NPC Modeling CER Task Forces. Questionnaire to assess relevance and credibility of modeling studies for informing health care decision making: an ISPOR-AMCP-NPC Good Practice Task Force report. Value Health 2014 Mar;17(2):174-82.

3: Bias assessment

Table 1. Bias assessment per study

Question	Pandharipande (9)	Pandharipande (10)	Peters (11)	Rulyak (12)	Koopmann (13)	Weinberg (15)	Cuchetti (16)	Raphel (17)
**1 – Validation**	High	High	High	High	Moderate	High	High	Moderate
**2 – Validation**	Low	Low	Low	Unclear	Moderate	High	Moderate	Moderate
**3 – Validation**	Low	Low	Low	Moderate	Low	Moderate	Moderate	Moderate
**4 – Design**	Low	Low	Moderate	Moderate	Low	High	Moderate	Moderate
**5 – Data**	High	High	High	High	High	High	High	High
**6 – Analysis**	Low	Low	Unclear	Moderate	Low	Moderate	Moderate	Moderate
**7 – Analysis**	Low	Low	Moderate	Low	Low	Low	Moderate	Low
**8 – Reporting**	Low	Low	Moderate	Low	Low	Moderate	High	Moderate
**9 – Interpretation**	Low	Low	Moderate	Moderate	Low	Low	Low	Low
**10/11 – conflict of interest**	Low	Low	Low	Low	Low	Low	Low	Low
